# Clustering of cardiovascular disease risk factors among first-year students at the University of Ibadan, Nigeria: a cross-sectional study

**DOI:** 10.1590/1516-3180.2021.0998.11052022

**Published:** 2022-08-29

**Authors:** Olumide Ebenezer Olufayo, Ikeoluwapo Oyeneye Ajayi, Samuel Osobuchi Ngene

**Affiliations:** I MD, MSc. Post-Master's Student, Department of Epidemiology and Medical Statistics, College of Medicine, University of Ibadan, Ibadan, Nigeria.; II MD, PhD. Professor, Department of Epidemiology and Medical Statistics, College of Medicine, University of Ibadan, Ibadan, Nigeria; Medical Consultant, Epidemiology and Biostatistics Research Unit, Institute for Advanced Medical Research and Training, College of Medicine, University of Ibadan, Ibadan, Nigeria.; University of Ibadan, College of Medicine, Institute for Advanced Medical Research and Training, Ibadan, Nigeria; III MD, MPH. Research Coordinator, Department of Epidemiology and Medical Statistics, College of Medicine, University of Ibadan, Ibadan, Nigeria; Research Office, Division of Cardiothoracic Surgery, Department of Surgery, University College Hospital, Ibadan, Nigeria; University College Hospital, Department of Surgery, Division of Cardiothoracic Surgery, Ibadan, Nigeria

**Keywords:** Cardiovascular diseases, Cluster analysis, Risk factors, Sedentary behavior, Clustering, Unhealthy diet, Physical inactivity

## Abstract

**BACKGROUND:**

Cardiovascular disease (CVD) is the second leading cause of death in sub-Saharan Africa. Globally, there is substantial evidence that modifiable risk factors for CVD are increasing in adolescents. Unfortunately, there is a paucity of information on the prevalence and clustering of these risk factors in adolescents.

**OBJECTIVES:**

This study explores the modifiable risk factors for CVD among first-year students at the University of Ibadan, Nigeria.

**DESIGN AND SETTING:**

This cross-sectional study was conducted at the University of Ibadan, Nigeria.

**METHODS:**

A total of 546 newly admitted students at the University of Ibadan, Nigeria, were recruited using stratified random sampling. An interviewer-administered questionnaire was used to obtain information from study participants between January and February 2016.

**RESULTS:**

The mean age of respondents was 19 ± 2.2 years with a male-to-female ratio of 1:1. The reported risk factors for CVD were smoking (1.6%), abdominal obesity (3.3%), alcohol consumption (3.7%), overweight/obesity (20.7%), unhealthy diet (85.3%), and physical inactivity (94.5%). Clustering of ≥ 2 risk factors was reported in 23.4% of students. Female students were twice as probably overweight/obese as male students (adjusted odds ratio [AOR] = 2.2; confidence interval [CI] = 1.41–3.43). Students whose fathers were skilled workers were 3.5 times more likely to be physically inactive (AOR = 1.7; CI = 0.97–2.96). The clustering of ≥ 2 risk factors was significantly higher among women and Muslims in bivariate analysis, whereas no significant association was found in multivariate analysis.

**CONCLUSIONS:**

Public health strategies to prevent CVD risk factors should begin in schools and extend to the entire community.

## INTRODUCTION

Cardiovascular disease (CVD) is a global public health problem and a leading cause of disability-adjusted life years in 2019.^1^ Most of these risk factors are caused by unhealthy lifestyles and habits; therefore, they are sometimes referred to as lifestyle risk factors and include smoking, tobacco, and excessive alcohol use, poor dietary patterns, and physical inactivity. Adolescents and young adults are particularly susceptible to these CVD risk factors in both developing and developed countries.^2,3^ Nearly all deaths from CVD occur among young people in Africa than in Europe and North America.^4^

Modifiable behaviors like physical inactivity, tobacco use, unhealthy diet and harmful alcohol consumption increase the risk of CVDs.^5^ About 38% of men and 40% of women aged at 18 years or older were overweight in 2014, and this figure is more than double the rate between 1980 and 2015.^4^ In Nigeria, the prevalence of overweight and obesity is 26.8% and 6.5%, respectively according to WHO.^6^ In southwestern Nigeria, a study revealed that only 60% of university undergraduates consumed the minimum recommended number of servings of grain (cereal) foods, while 60%, 85%, and 40% of students did not meet the recommended daily allowance for protein, calcium, and iron respectively.^7^

Globally, 23% of men and 32% of women over the age of 18 years were insufficiently physically active in 2016.^8^ Not having sufficient physical activity is one of the ten leading risk factors for global mortality. These people have at 20%–30% increased risk in all-cause mortality compared with those who engage in at least 150 minutes in moderate-intensity physical activity per week, or equivalent, as recommended by the World Health Organization.^9^ Physical inactivity causes 6% in the burden of disease from coronary heart disease, 30% of ischemic heart disease, 7% of type 2 diabetes, 10% of breast cancer, and 10% of colon cancer.^9^

Excessive fat accumulation produces an accumulation of lipids around the visceral adipose tissue, which is another risk factor for developing CVDs.^10^ A study also shows that the prevalence of abdominal obesity was low among young adults in a tertiary institution.^11^ A study among Nigerian university students found a higher proportion of abdominal obesity (5.9%) among female undergraduate students compared with their male counterparts (0.8%)^12^

The clustering of CVD risk factors has an amplifying effect that induces increased CVD risk.^13,14^ These risk factors can be observed in early adolescence and continue into adulthood.^15^ Multiple clustering of these risk factors in adolescents and young adults leads to an initial stage of CVD such as atherosclerosis.^13^ The accumulation of cholesterol, lipids and fibrous plagues begins in arterial walls at the age of 10 years and increases over time until it manifests overtime and manifests as an atherosclerotic lesion in adulthood.^13,14^ Therefore, tracking of the clustering of multiple CVD risk factors is highly essential and is a sine qua non for mitigating the threat of CVD in adolescents and young adults.

Clustering of CVD risk factors among young people has been well explored in the literature, with interesting findings in low-, middle-, and high-income countries.^16–19^ However, there is a paucity of information on this subject matter among university students in Nigeria, particularly newly admitted students who will most likely experience a significant change in their lifestyle. Therefore, this study examined the risk factors for CVD and their clustering in first-year undergraduate students at the University of Ibadan.

## OBJECTIVE

Against this background, this study investigated CVD risk factors and their clustering in first-year undergraduate students at the University of Ibadan.

## METHODS

### Study site

The University of Ibadan has 13 faculties and enrolls at least 3,000 students annually. The University of Ibadan maintains a well-rounded program of sport and athletic activities on campus under the supervision of the Director of Sports. Aside from maintaining a sound body, which is beneficial for progressive thinking and rigorous academic pursuits, students have the added benefit of being exposed to modern facilities and techniques through active participation in various sports.

### Study design and population

This was a cross-sectional study among the first-year students of the 2014/2015 academic year at the University of Ibadan, Oyo State, Nigeria. All consenting first-year students at the University of Ibadan aged 15–35 years were eligible to participate in the study while those with physical deformities were excluded.

### Sample size and sampling procedure

The sample size was calculated using the Leslie-Kish formula, representing 23.7% of adolescents with a cluster of three CVD risk factors,^3^ and a sampling error of 5%. A stratified random sampling technique was used to recruit eligible respondents. The University of Ibadan has academic programs in 13 faculties. Out of the nine faculties, six faculties were randomly selected while all faculties in the College of Medicine, University of Ibadan were selected for the study. In each randomly selected faculty, 50% of the departments were considered except in the Faculty of Dentistry and Public Health, where only one department was chosen while in Clinical Sciences, 100% of the departments were admitted for the 2014/2015 academic session were used. The total number of first-year students (study population) in the randomly selected departments was determined. Then, a proportional allocation of the sample was carried out to determine the number of first-year students in each department. Then, systematic random sampling was used to select the study participants (students) from each department based on the sampling interval. Each person (student) in each department was then assigned a number, and each Kth person was taken from the total number of first-year undergraduate students in each randomly selected department, and the starting point was randomly selected.

### The data collection instrument

A semi-structured questionnaire was used to obtain information on the socio-demographic, anthropometric, and lifestyle characteristics of the respondents. Data were collected from January 2016 to February 2016. The questionnaire was validated by experts and then tested among 20 first-year students at another faculty that was not selected for the study. A Cronbach's alpha of 0.8 was obtained. These students had a similar age range to the study participants.

Scale and meter rules, respectively, measured weight and height. The waist circumference (WC) of each participant was measured with a nonelastic tape measure. WC was measured midway between the lowest rib and the superior border of the iliac crest at the end of normal exhalation to the nearest 0.1 cm.^6^

The validated International Physical Activity Questionnaire Short Form (IPAQ-SF) was used to measure students' level of physical activity. Respondents with less than 600 metabolic equivalent minutes of work/week were classified as not physically active.^20^ Respondents with body mass index ≥ 29.9 kg/m^2^ were classified as overweight/obese.^21^

Respondents with waist circumference greater than or equal to 88 cm (women) and greater than or equal to 102 cm (male) were classified as abdominally obese.^21^

Dietary patterns were assessed using eating habit questionnaires. Respondents who consumed fewer than five servings of fruits and vegetables per day on at least five days per week were classified as having an unhealthy diet.^22^

Alcohol consumption of more than three standard units/day for men or more than two standard units/day for womenwas classified as excessive alcohol consumption.^23^ Current Smoking status was measured as use of tobacco (smoke and/or smokeless) within the past month.^24^

### Data analysis

Data were entered and analyzed using SPSS version 24 (IBM Corp. Released 2016. IBM SPSS Statistics for Windows, version 24.0. Armonk, New York: IBM Corp). The general characteristics of the respondents are presented using descriptive statistics. Factors associated with CVD risk factors and their clusters (≥ 2) were assessed using the chi-square test. Binary logistic regression was used to analyze CVD predictors considering a CI of 95%. The significance level was set at P < 0.05.

### Ethical considerations

This study was approved by the Ethics Committee of the of Ibadan on October 23, 2015 under the approval number: NHREC/05/01/2008a. The chairman of this committee can be contacted at the Biode Building, Room 210, 2nd Floor, Institute for Advanced Medical Research and Training, College of Medicine, University of Ibadan. e-mail: uiuchirc@yahoo.com and, uiuchec@gmail.com.

## RESULTS

A total of 546 first-year students (first-year students) participated in the survey. Table 1 shows that most respondents (81.7%) were between 20 years old and younger, while the mean age of the respondents was 19 ± 2.2 years. Table 1 shows the socio-demographic characteristics of the study participants. More than half of the respondents were female (55.1%) and the majority (99.3%) were single. Christianity (86.1%) was the predominant faith. Most students (93.0%) lived in university dormitories. The majority (49.6%) of the participants had fathers who held skilled occupations, others (38.3%) had fathers who held semi-skilled occupations, and a few (12.1%) of the respondents' fathers had unskilled occupations. About (62.5%) of the respondents received monthly allowances between N10,001 and N20,000, while (31.3%) received monthly allowances between N10,000 and below.

**Table 1:** Socio-demographic characteristics and cardiovascular disease risk factor clustering among newly admitted undergraduate students of the University of Ibadan, Nigeria (n = 546)

Variables		Frequency	Percent (%)
**Gender**			
	Male	245	44.9
	Female	301	55.1
**Age group (years)**			
	≤ 20	446	81.7
	≥ 21	100	18.3
**Marital status**			
	Single	542	99.3
	Married	4	0.7
**Religion**			
	Christianity	470	86.1
	Islam	76	13.9
**Residence**			
	University hostel	508	93.0
	Off campus	38	7.0
**Fathers’ occupation**			
	Skilled	271	49.6
	Semi-skilled	209	38.3
	Unskilled	66	12.1
**Mothers’ occupation**			
	Skilled	277	50.7
	Semi-skilled	236	43.2
	Unskilled	33	6.0
**Monthly allowance (**N**)**			
	≤ 10,000	171	31.3
	10,001-20,000	341	62.5
	≥ 20,001	34	6.2
**Cardiovascular disease risk factors**			
	Overweight/obese	113	20.7
	Unhealthy diet	466	85.3
	Currently smoking	9	1.6
	Physical inactivity	516	94.5
	Abdominal obesity	18	3.3
	Alcohol use	20	3.7
	Clustering risk factors (≥ 2)	128	23.4

**Figure 1:**
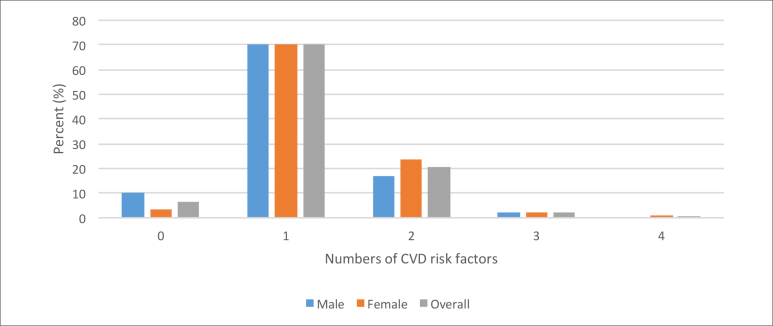
Clustering of cardiovascular diseases (CVD) risk factors among newly admitted undergraduate students at the University of Ibadan, Nigeria.

The various CVD risk factors and their clustering are shown in Table 1. These included current smoking (1.6%), abdominal obesity (3.3%), alcohol consumption (3.7%), overweight/obesity (20.7%), unhealthy diet (85.3%), and physical inactivity (94.5%), whereas 23.4% had at least two of these CVD risk factors. Figure 1 shows the number of CVD risk factors and their clustering by sex. Most respondents had one CVD risk factor (70.1%), followed by two risk factors (20.7%), three risk factors (2.4%), and four risk factors (0.4%); 6.4% had none of the risk factors studied.

Table 2 shows the bivariate analysis of the factors associated with CVD risk factors and their clusters. The clustering of CVD risk factors was significantly higher in women than in men (P < 0.05) and higher in Christians than in Muslims (P < 0.05). Table 3 shows the multivariate analysis of the predictors of CVDs and their clusters. Women were twice as likely as male respondents to be overweight/obese (adjusted odds ratio, AOR = 2.2; 95% CI = 1.41–3.43; P value = 0.001). Muslims were 5.1 times more likely to smoke than Christians (AOR = 5.1; 95% CI = 1.32–19.37; P value = 0.018). Respondents whose parents were skilled workers were 3.5 times more likely to be physically inactive than respondents whose parents were unskilled workers (AOR = 3.5; 95% CI = 1.24–9.85; P value = 0.018).

**Table 2:** Bivariate analysis of the risk factors of cardiovascular diseases and its clustering among newly admitted undergraduate students at the University of Ibadan, Nigeria

		Overweight/obesity % (95% CI)	Unhealthy diet % (95% CI)	Smoking % (95% CI)	Physical inactivity % (95% CI)	Abdominal obesity % (95% CI)	Alcoholic consumption % (95% CI)	Clustering of risk factors (≥ 2) % (95% CI)
**Gender**								
	Male	13.9 (0.21–0.74)	86.1 (0.67–1.79)	0.8 (0.08–2.51)	6.5 (0.92–5.64)	0.3 (0.01–0.05)	4.5 (0.77–5.52)	19.6 (0.01–0.03)
	female	26.2 (1.35–4.75)	84.7 (0.56–1.48)	2.3 (0.04–0.19)	4.7 (0.04–0.16)	6.0 (0.12–0.36)	3.0 (0.04–1.16)	26.6 (0.01–0.06)
	P for trend	< 0.001*	0.644	0.168	0.338	< 0.001*	0.353	< 0.001*
**Age (in years)**								
	≤ 20	20.9 (0.62–2.93)	86.8 (0.95–2.99)	1.6 (0.15–5.38)	5.6 (0.49–4.17)	2.9 (0.09–0.89)	3.1 (0.21–1.72)	22.9 (0.46–1.50)
	≥ 21	20.0 (0.34–1.61)	79.0 (0.33–0.54)	2.0 (0.05-1.04)	5.0 (0.08–1.66)	5.0 (1.16–1.61)	6.0 (0.18–1.33)	26.0 (0.66–2.16)
	P for trend	0.849	0.047*	0.760	0.810	0.220	0.169	0.504
**Religion**								
	Christianity	19.8 (0.42–2.29)	86.2 (0.77–2.79)	1.1 (0.06–1.47)	5.1 (0.25–2.10)	3.2 (0.33–3.89)	3.0 (0.06–0.12)	19.0 (0.04–0.19)
	Islam	26.3 (0.44–2.37)	80.3 (0.36–1.29)	5.3 (0.14–1.50)	7.9 (0.17–1.19)	3.9 (0.07–1.82)	7.9 (1.23–1.69)	4.4 (0.03–0.30)
	P for trend	0.192	0.177	0.008*	0.322	0.732	0.034*	0.020*
**Residence**								
	University hostel	18.9 (0.11–1.11)	85.8 (0.60–3.26)	1.8 (0.01–2.08)	5.9 (0.45–10.86)	3.3 (0.18–5.51)	3.7 (0.19–4.45)	22.0 (0.01–1.04)
	Off campus	1.8 (0.90–8.57)	78.9 (0.30–1.65)	0.4 (0.01–1.23)	1.2 (0.01–0.45)	2.6 (0.02–1.87)	2.6 (0.02–1.88)	1.5 (0.10–1.49)
	P for trend	0.375	0.247	0.408	0.123	0.812	0.726	0.366
**Father's occupation**								
	Skilled job	23.2 (0.56–4.34)	87.5 (0.49–2.59)	1.5 (0.03–5.98)	3.3 (0.03–0.45)	3.0 (0.43–12.73)	3.7 (0.12–1.48)	12.5 (0.14–0.42)
	Semi-skilled	18.2 (0.43–3.49)	82.3 (0.31–1.61)	2.4 (0.03–8.58)	6.7 (0.13–1.38)	4.3 (0.14–5.64)	2.4 (0.06–1.99)	8.1 (0.01–0.16)
	Unskilled	18.2 (0.40–1.52)	86.4 (0.27–2.23)	0.4 (0.08–1.59)	10.6 (0.21–0.83)	1.5 (0.14–2.67)	7.6 (0.05–0.38)	2.9 (0.57–2.23)
	P for Trend	0.344	0.276	0.393	0.041*	0.490	0.148	0.237
**Mother's occupation**								
	Skilled job	22.7 (0.37–5.40)	84.5 (0.26–2.23)	1.4 (0.03–1.34)	4.7 (0.06–1.55)	4.3 (0.12–5.64)	3.2 (0.15–4.31)	11.9 (0.01–1.15)
	Semi-skilled	17.8 (0.23–3.44)	86.4 (0.37–3.13)	1.7 (0.03–0.30)	5.9 (0.06–1.68)	2.1 (0.04–2.34)	3.8 (0.21–5.23)	9.7 (0.06–1.49)
	Unskilled	24.2 (0.33–1.20)	84.8 (0.82–2.38)	3.0 (0.028–0.30)	9.1 (0.10–1.49)	0.2 (0.02–1.40)	6.1 (0.08–1.39)	1.8 (0.83–3.31)
	P for trend	0.338	0.819	0.793	0.535	0.374	0.709	0.609

* Statistically significant at P < 0.05.

**Table 3:** Multivariate analysis of the risk factors of cardiovascular diseases and its clustering among newly admitted undergraduate students at the University of Ibadan, Nigeria

Variables			AOR	95% CI (AOR)		P value
				Lower	Upper	
**Overweight/Obese**						
	**Gender**					
		Male (ref)	1.0			
		Female	2.2	1.41	3.43	0.001*
	**Religion**					
		Christianity (ref)	1.0			
		Islamic	1.4	0.80	2.51	0.220
**Dietary pattern**						
	**Age (years)**					
		≤ 20 (Ref)	1.0			
		≥ 21	1.7	0.97	2.96	0.061
	**Religion**					
		Christianity (ref)	1.0			
		Islamic	1.5	0.78	2.75	0.229
**Smoking**						
	**Gender**					
		Male (ref)	1.0			
		Female	2.8	0.57	13.75	0.202
	**Religion**					
		Christianity (ref)	1.0			
		Islamic	5.1	1.32	19.37	0.018*
**Physically inactive**						
	**Residence**					
		University hostel (ref)	1.0			
		Off campus	2.3	0.48	10.88	0.298
	**Fathers’ occupation**					
		Unskilled (ref)	1.0			
		Semi-skilled	2.1	0.88	4.96	0.090
		Skilled	3.5	1.24	9.85	0.018*
**Clustering of CVD risk factor (≥ 2)**						
	**Gender**					
		Male (ref)	1.0			
		Female	1.5	0.98	2.22	0.061
	**Religion**					
		Christianity (ref)	1.0			
		Islamic	1.6	0.95	2.74	0.080

* Statistically significant at P < 0.05; AOR = adjusted odds ratio, ref = reference.

## DISCUSSION

To our knowledge, our study is one of the first to explore the clustering of CVD risk factors in newly admitted students in this part of the continent. We found that clustering of two CVD risk factors was observed in one-fifth of the students. The most common of these risk factors were physical inactivity, unhealthy diet, and overweight/obesity, whereas alcohol consumption, smoking, and abdominal obesity were rare in our study population.

The high response rate (98.0%) observed in this study is consistent with similar studies in Nigeria., ^25^ and Ghana.^12^ The proportion of women who participated in this study was higher than that of male respondents. The female predilection in our study corresponds with the reports of Ekerand colleagues among high school students in Turkey.^26^ and a national survey of students in various tertiary institutions between 2010 and 2015 in Nigeria.^27^

An unhealthy dietary pattern was evident among undergraduate students in this study, which corresponds to previous studies in Nigeria^28,29^ A higher rate of unhealthy dietary lifestyle among women supports the report by Omage and Omuemu.^29^

In line with the report on students in Bangladesh,^30^ we found that students who lived off campus had poorer dietary patterns than students who lived in a university dormitory. One plausible reason for this is that students who live off campus prepare their own food, which is better than what is available in school cafeterias. Others live with family members or relatives who prepare the food for them.

The prevalence of current smoking was low in our study (1.6%), compared with previous studies among adolescents and young adults that found 6.8% in Ethiopia,^31^ We found that students who lived off campus had lower dietary behaviors than students who lived in a university dormitory. One plausible reason is that students living off campus prepare their own food, which is better than what is available in school cafeterias. Others live with family members or relatives who prepare the food for them.

The prevalence of current smoking in our study was low (1.6%), compared with previous studies among adolescents and young adults that found 6.8% in Ethiopia,^32^ 9.0% in Oman,^33^ 11.1% in New Zealand^34^ and 27.9% in Turkey.^35^ The low prevalence of smoking observed in our study is likely due to risky behaviors such as smoking are reportedly more common among students in higher grades students than newly admitted students.^7^ Muslims were more likely to smoke than Christians in our study, which contradicts the report by Hussain and colleagues.^36^ Nonetheless, the teachings of both religions have been reported to influence the behavior of their believers and to condemn smoking and alcohol consumption.^37,38^ Some authors have argued that people tend not to disclose their correct smoking status despite assurances of confidentiality of data collected.^39^ Hence, the reported smoking status should be interpreted with this in mind.

This study also show that a small proportion of the study population was highly engaged in physical activity. This is very similar to the findings in the study by Eleojo et al., who proved that only a small proportion of the study population was physically active.^40^ A previous multicentre study revealed that a proportion of male respondents were physically inactive compared with female respondents,^40^ which corresponds to our findings. Our study found an association between physical inactivity and father's occupation. This supports the assertion that parental factor influences the level of physical activity of their children. Parents' occupation and type of living environment have been seriously implicated.^41,42^ The low prevalence of obesity in our study is in contrast to the report of Sabageh and Ojofeitimi with higher prevalence.^43^ A study also showed that the prevalence of abdominal obesity was high in the study population.^11^ A study showed that the prevalence of abdominal obesity as determined by the waist circumference, was higher in male respondents than in female respondents.^44^ This present study also revealed that abdominal obesity is significantly related to gender of which male respondents have a higher proportion of central obesity than female respondents.

This study revealed a low prevalence of alcohol intake among the study population. Another study was done by Alex-Hart and colleagues showed that the prevalence of alcohol consumption was 28.6% significantly higher prevalence from this study.^45^ Another study reported a higher proportion of alcohol consumption among males students compared to their female colleagues.^46^ Several studies revealed that excessive alcohol consumption is much more common among undergraduate students who reside in the university hostels away from their permanent domicile. This is very similar to the findings reported in this study which shows that students who live on campus had a higher proportion of alcohol intake compared to those who live off campus.^47^ This study revealed that most of the respondents had at least two risk factors. This study also corresponds to another study done by Olawuyi and Adeoye, which revealed that a higher proportion of the population had at least two non-communicable disease risk factors.^48^ The clustering risk factors have been associated with a higher risk of developing CVDs.^49^ A study conducted among young adults in southwest Nigeria reported that there is no significant difference in clustering risk factors for CVDs between the males and females who participated in the study, which is in contrast with the finding of this study. Another study conducted among university students in Libya revealed that there was a significant relationship between clustering risk factors and socio-demographic characteristics of university students.^50^ A study conducted among young adults in Yaoundé, Cameroon revealed that the prevalence of some major CVD risk factors increase due to a lack of a appropriate behavioral approach towards healthy living.^51^ A previous study also showed a higher proportion of obesity among the females' respondents compare to the male respondents.^52^ It is vital to know the relationship between socio-demographic characteristics such as age, gender, and clustering risk factors for CVDs explicitly because it will help to control and prevent CVDs especially among undergraduate students.^53^ A previous study revealed that male undergraduate students had lower awareness of the clustering risk factors to CVD compared to their female counterparts.^54^ A study conducted among Nigeria undergraduate students revealed that there was no significant difference between the risk factors for CVD among the gender stratification.^55^ The findings of our study suggest there is urgent need for public health strategies that will improve physical activity and consumption of healthy diets. This should be done in corroboration with the university management.

### Implications of the findings of the study

Note that most young adults do not take care of their health before coming to university. The missed opportunities that result from poor health facilities for young people could be addressed at the university where health facilities exist, through the approach prevention strategy developed by Leavell:

- Primary prevention: adding physical activity to the academic calendar will improve physical fitness.
- Primary prevention: every restaurant in the university community offers fruit for consumption by students after meals. This will also encourage students to consume less high cholesterol foods.
- Secondary prevention: screening and treatment (i.e., Dietary changes, exercise, behavior modification, and prescription of weight loss medications).
- Tertiary prevention: this stage is important for the management and control of obesity in obese/overweight students by attending school-based sports facilities specialized in exercise, such as the gymnasium.

### Strengths and limitations

The strength of our study was that it was interviewer-administered, which explains the high response rate and limited missing data. Nevertheless, like any other cross-sectional study, our study shows an association and not a causal relationship. Additionally, our study investigated the level of clustering in a selected university in Ibadan, Nigeria. Additionally, this study focused on first-year students from the selected departments. Therefore, these results may not be generalizable to other universities in Ibadan or Nigeria.

## CONCLUSION

Our study's clustering of cardiovascular risk factors was unexpectedly high, with high levels of physical inactivity and an unhealthy diet. The results of this study underscore several issues that need to be considered in reducing the risk of CVD in first-year students at the University of Ibadan.
